# Formation mechanism of SiGe nanorod arrays by combining nanosphere lithography and Au-assisted chemical etching

**DOI:** 10.1186/1556-276X-7-140

**Published:** 2012-02-18

**Authors:** Chih-Chung Lai, Yun-Ju Lee, Ping-Hung Yeh, Sheng-Wei Lee

**Affiliations:** 1 Institute of Materials Science and Engineering, National Central University, Jhongli, 32001, Taiwan; 2 Department of Physics, Tamkang University, Danshui District, New Tapei, 25137, Taiwan

**Keywords:** Ge, nanorod, self-assembly, nanosphere lithography

## Abstract

The formation mechanism of SiGe nanorod (NR) arrays fabricated by combining nanosphere lithography and Au-assisted chemical etching has been investigated. By precisely controlling the etching rate and time, the lengths of SiGe NRs can be tuned from 300 nm to 1 μm. The morphologies of SiGe NRs were found to change dramatically by varying the etching temperatures. We propose a mechanism involving a locally temperature-sensitive redox reaction to explain this strong temperature dependence of the morphologies of SiGe NRs. At a lower etching temperature, both corrosion reaction and Au-assisted etching process were kinetically impeded, whereas at a higher temperature, Au-assisted anisotropic etching dominated the formation of SiGe NRs. With transmission electron microscopy and scanning electron microscopy analyses, this study provides a beneficial scheme to design and fabricate low-dimensional SiGe-based nanostructures for possible applications.

## Introduction

Over the past few decades, intensive research efforts have been devoted to the fabrication and characterization of Si-based nanostructures due to their intrinsic physical properties, high packing density, and compatibility with current Si technology [1]. Self-assembled Si-based nanostructures are of particular interest because self-assembly provides a possible way to realize nanostructures without process-induced damages, which are frequently observed in the samples defined by electron (e)-beam lithography or reactive ion etching (RIE) [2,3]. Ge/Si has become a model system for the fabrication and investigation of nanometer-scale heteroepitaxy due to their moderate lattice mismatch (4.2%) [4,5]. The fabrication of SiGe nanowire arrays is one of the most interesting topics [6,7]. Recently, the use of Si-based nanowires as high-performance devices or sensors has been extensively reported [8-12]. There are several methods to fabricate nanowire structures, such as e-beam lithography [13] and vapor-liquid-solid growth [14-16], and metal-assisted chemical etching [17-20]. Previous works have demonstrated that nanosphere lithography (NSL) provides an efficient way to fabricate self-organized, ordered, and close-packed sphere arrays [21,22]. However, there have been few studies paying attention on the formation mechanism of SiGe NRs. In this work, we fabricated SiGe NR arrays by combing NSL and Au-assisted chemical etching. The influences of chemical etching conditions on the morphologies of as-etched SiGe NRs were investigated to clarify their formation mechanism.

## Experimental details

**Figure 1:**
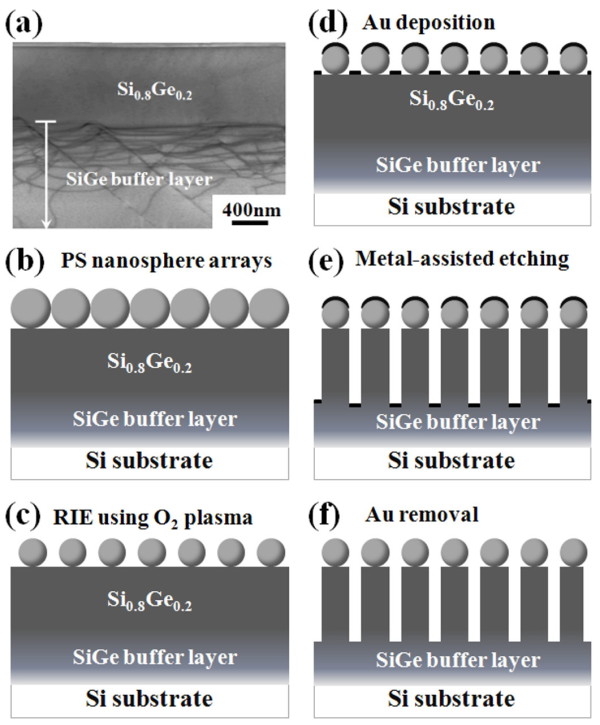
**Schematic diagram of fabrication process of SiGe NRs**. (**a**) TEM image of 1-μm-thick Si_0.8_Ge_0.2 _epilayer with the SiGe buffer layer grown on Si wafer. (**b**) PS nanospheres in diluted colloidal form were drop-placed onto the freshly prepared hydrophilic SiGe substrate. (**c**) The sizes of the PS spheres were reduced by RIE treatment. (**d**) A 20-nm-thick Au film was deposited by e-beam evaporation. (**e**) SiGe NRs were formed by Au-assisted wet chemical etching. (**f**) Finally, Au film is removed by an Au etchant.

The schematic depiction of experimental procedures is shown in Figure 1. *p*-Type (001)-oriented Si wafers 10 to 25 Ω cm in size and 100 mm in diameter were used in the present study. All SiGe heterostructures used in this study were grown at 550°C in a multi-wafer ultra-high vacuum chemical vapor deposition (UHV/CVD) system. Before epitaxial growth, the Si wafers are dipped in a 10% HF solution to achieve the hydrogen-passivated surface and then transferred into an UHV/CVD system. A 50-nm-thick Si buffer layer was first grown and followed by growth of a 2-μm-thick SiGe buffer layer and a 1-μm-thick Si_0.8_Ge_0.2 _uniform epilayer, as shown in Figure 1a. The wafer with the SiGe epilayer was sliced into 1 × 1 cm^2 ^as the templates. Next, polystyrene (PS) nanospheres in diluted colloidal form were then drop-placed onto the freshly prepared hydrophilic substrate, as illustrated in Figure 1b. An area of a monolayer of polystyrene nanospheres then forms upon complete water evaporation under ambient condition. Subsequently, RIE process using O_2 _plasma with a power of 30 W was employed to reduce the sizes of the PS nanospheres, as illustrated in Figure 1c. In this step, the PS nanospheres with a reduced size formed non-closely packed arrays on the surface. Next, a 20-nm-thick gold film was then deposited onto the substrate by e-beam evaporation, as illustrated in Figure 1d. Due to the PS monolayer mask, an Au film with patterned nanohole arrays was formed. The patterned Au thin film acts as a catalyst in the following Au-assisted etching process. The samples were then dipped into the freshly prepared etching solution (2:1:2:5 (*v*/*v*) HF/H_2_O_2_/C_2_H_5_OH/DI water mixture) to form SiGe NR arrays under various etching conditions, as shown in Figure 1e. The diameter, spacing, and density of SiGe NRs should be defined by the starting reduced size of PS nanospheres. Once SiGe NRs were formed, the Au metal was washed away using an Au etchant (3:1 (*v*/*v*) HCl/HNO_3 _mixture), leaving PS nanospheres on the surface as labels for observing the etched SiGe NRs. Finally, the morphologies and microstructures of the resulting SiGe NRs were characterized by scanning electron microscopy (SEM) and transmission electron microscopy (TEM) in conjunction with an energy dispersion spectrometer (EDS).

## Results and discussion

**Figure 2:**
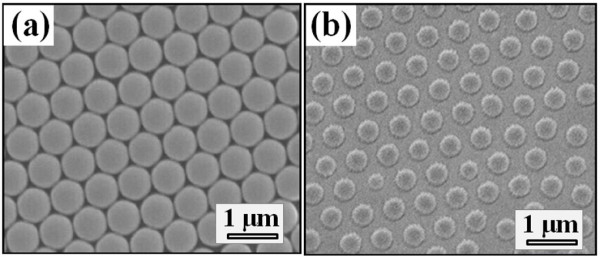
**Top-view SEM images of PS nanospheres**. (**a**) Before and (**b**) after the RIE treatment using O_2 _as precursor gas.

Top-view SEM images of self-assembled PS nanosphere arrays before and after RIE treatment are shown in Figure 2a, b, respectively. Prior to the RIE treatment, the PS nanospheres were closely packed on the SiGe substrate with a uniform diameter of 600 nm. After the RIE treatment, the PS nanospheres were then trimmed to an average size of 420 nm. These reduced sizes of PS nanospheres were also found to be deformed to a truncated shape, possibly due to the thermal heating effect during the O_2 _plasma RIE process.

**Figure 3:**
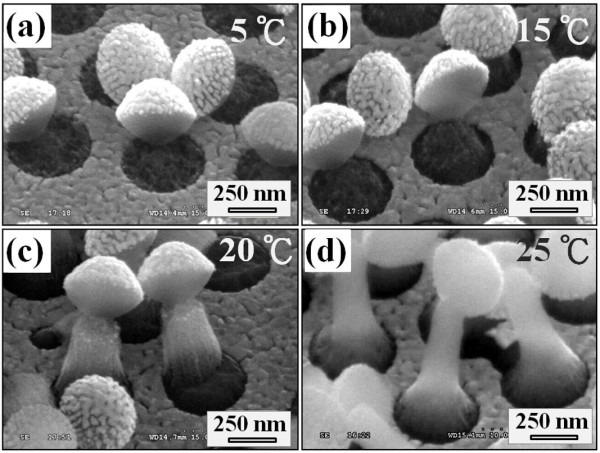
**SEM images of SiGe NRs fabricated by Au-assisted wet chemical etching at various solution temperatures**. Solution temperatures of (**a**) 5°C, (**b**) 15°C, (**c**) 20°C, and (**d**) 25°C for 20 min.

Figure 3 shows the temperature dependence of the morphologies of SiGe NR arrays etched at temperatures from 5°C to 25°C. The etching time was fixed at 20 min for all SiGe NRs. By the fact that PS nanospheres are on top of the etched structure, we can verify that the etched SiGe nanostructures are composed of the as-grown SiGe epilayer. At lower etching temperatures (5°C to 15°C) as shown in Figure 3a, b, the etched SiGe nanostructures show a necklike body with a thin diameter underneath the truncated PS nanospheres. The maximal height of the etched SiGe nanostructures was limited to be about 300 nm. However, by increasing the etching temperature to 20°C and 25°C, the etched SiGe nanostructures became apparently longer (about 1 μm at 25°C), i.e., the formation of SiGe NRs. These results demonstrated that the morphologies of etched SiGe nanostructures are strongly influenced by the etching temperatures and potentially can be controlled by varying other etching conditions. Herein, we propose a mechanism involving a locally temperature-sensitive redox reaction to explain this strong temperature dependence of the morphologies of the etched SiGe NRs.

It is well known that metal-assisted chemical etching in the H_2_O_2_/HF solution may occur as a localized electrochemical process, with the nanometer-sized metal acting as a local cathode and microscopically local Si acting as an anode [17]. Their corresponding half-cell reaction can be outlined as the following.

$$\begin{array}{ccc} \text{Cathode}\;\text{reaction}: & {\text{H}}_{2}{\text{O}}_{2}+2{\text{H}}^{+}\to 2{\text{H}}_{2}\text{O}+\text{2}h^{+}, & \\ & 2{\text{H}}^{+}+2e^{-}\to {\text{H}}_{2}↑. \end{array}$$

$$\begin{array}{ccc} \text{Anode}\;\text{reaction:} & \text{Si}+2{\text{H}}_{2}\text{O}\to \text{Si}{\text{O}}_{2}\left(\text{s}\right)+4{\text{H}}^{+}+4e^{-} & \\ & \text{Si}{\text{O}}_{2}\left(\text{s}\right)+6\text{HF}\to {\text{H}}_{2}\text{Si}{\text{F}}_{6}+2{\text{H}}_{2}\text{O}\text{.} \end{array}$$

**Figure 4:**
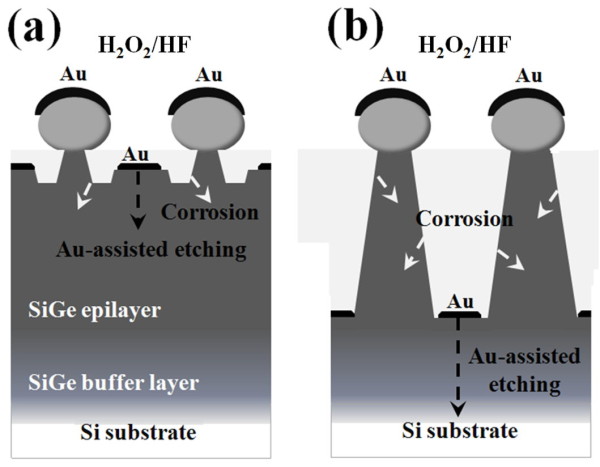
**Schematic of the formation of SiGe nanostructures**. These are formed by Au-assisted chemical etching at relatively (**a**) low and (**b**) high solution temperatures.

Considering the SiGe/Au/H_2_O_2_/HF system in this study, because Au is more electronegative than SiGe materials, the deposited Au film strongly attracts electrons from the underlying SiGe substrate and becomes negatively charged [23]. The deposited Au film serves to catalyze the subsequent reduction of H_2_O_2 _and H^+ ^ions and thus facilitates the oxidation of the underlying SiGe. Therefore, once Au-assisted chemical etching dominates the whole etching process, an anisotropic etching can be expected. On the other hand, there have been many studies reporting that SiGe materials tend to be more vulnerable to the H_2_O_2_/HF solutions than pristine Si wafer [6,24]. This means that even if there is no Au catalyst existing, SiGe may still suffer 'attacking' from the H_2_O_2_/HF solution, i.e., the corrosion reaction, which is principally an isotropic etching process. Therefore, as illustrated in Figure 4, there exist two etching mechanisms competing for the formation of SiGe nanostructures by Au-assisted chemical etching. As the etching temperature is low, both the corrosion reaction and Au-assisted etching process are kinetically impeded. Thus, necklike etched nanostructures with a limited height could be observed (Figure 4a). By increasing the etching temperature above 20°C, more temperature-sensitive Au-assisted anisotropic etching begins to dominate the whole etching process, and SiGe NRs form (Figure 4b). Nevertheless, isotropic corrosion reaction still proceeds in the meantime. Therefore, all SiGe NRs have a taper-like shape with a diameter less than that defined by the PS nanospheres (420 nm); that is also why all SiGe nanostructures in this study have a base horizontally lower than the surrounding Au film. It is also worthwhile noting that if we increase the temperature above 40°C, only straight Si nanowire arrays would be obtained since the upper SiGe parts have been etched away (not shown here). This is because both the corrosion reaction and Au-assisted etching rates are significantly enhanced at such a high temperature.

**Figure 5:**
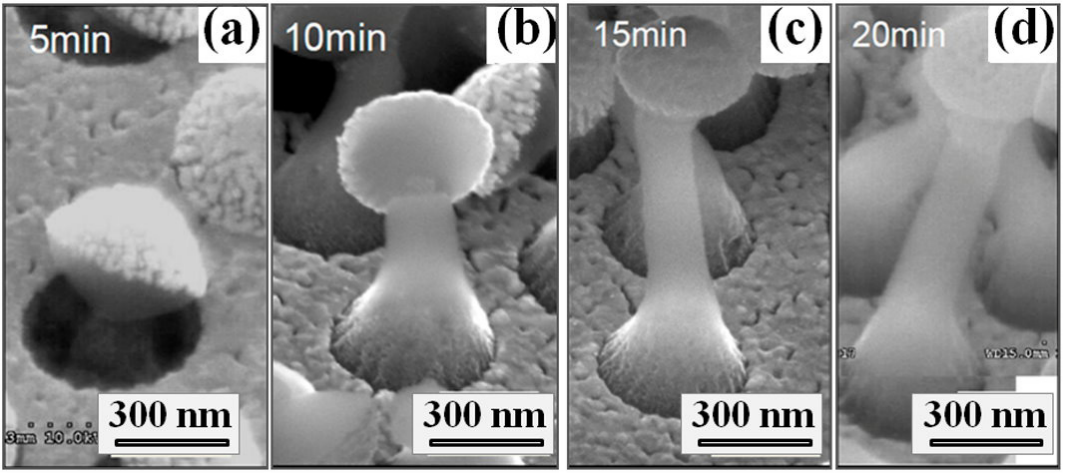
**SEM images of SiGe NRs etched at a solution temperature at various times**. SEM images of SiGe NRs etched at solution temperature of 25°C for (**a**) 5, (**b**) 10, (**c**) 15, and (**d**) 20 min.

By fixing the etching temperature at 25°C, we can further observe the formation evolution of the SiGe NRs. As seen in Figure 5a, the etched SiGe structures started with a necklike shape, which is very similar to that appearing at low etching temperature. It is speculated that Au-assisted chemical etching process may be hindered at the initial stage of etching possibly because the initial oxidation of SiGe underneath the Au film is limited by the reactant transport. After that, the SiGe NRs increase their lengths with the increasing etching time, as shown in Figure 5b, c, d. Note that SiGe NRs with etching times of 15 min and 20 min have similar rod lengths (about 1 μm). We infer that the Au-assisted etching rate would be slowed down as the SiGe buffer layer is reached, where the Ge composition decreases gradually into the depths.

**Figure 6:**
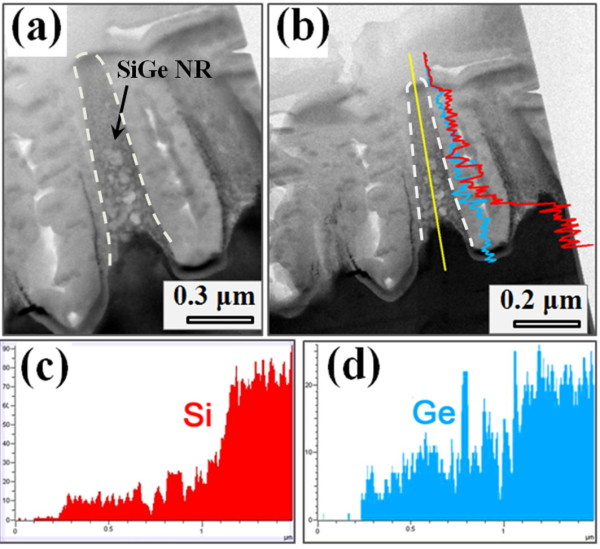
**TEM image of a SiGe NR and compositional distribution of Si and Ge**. (**a**) Cross-sectional TEM image of a SiGe NR, which is highlighted by a dotted line. (**b**) The composition of the SiGe NR can be characterized by the EDS line scan, where the Si and Ge compositional distribution are shown in (**c**) and (**d**), respectively.

The TEM-related data of SiGe NRs are gathered in Figure 6. Figure 6a shows a cross-sectional TEM image of a typical SiGe NR. No dislocation or any residual reactant was observed in the SiGe NR structure. With the EDS analysis, we can further confirm that SiGe NRs are composed of SiGe materials, i.e., they are a replica of the as-grown SiGe epilayer. Low-dimensional SiGe nanostructures have many potential applications, such as chemical and biochemical sensing [25,26]. Notably, compared with Si materials, SiGe alloys have a tunable and lower work function, which is an important factor for designing field electron emitters [27]. Therefore, by optimizing the microstructural parameters, like the tip curvature and aspect ratio, taper-like SiGe NRs formed by Au-assisted chemical etching may promise to be applicable for fabricating field emitters. Further work remains under investigation.

## Conclusions

In this study, the formation mechanism of SiGe NR arrays fabricated by combining NSL and Au-assisted chemical etching has been investigated. By precisely controlling the etching rate and time, the lengths of the SiGe NRs can be tuned. The morphologies of SiGe NRs changed dramatically by varying the etching temperatures. We propose a mechanism involving a locally temperature-sensitive redox reaction to explain this strong temperature dependence of the morphologies of SiGe NRs. At a lower etching temperature, both corrosion reaction and Au-assisted etching process were kinetically hindered, whereas at a higher temperature, Au-assisted anisotropic etching dominated the formation of SiGe NRs. With TEM and SEM analyses, this study provides a beneficial scheme to design and fabricate low-dimensional SiGe-based nanostructures for possible applications.

## Competing interests

The authors declare that they have no competing interests.

## Authors' contributions

C-CL carried out the nanorod experiments and data analysis under the instruction of S-WL. Y-JL and P-HY performed the TEM measurements. All the authors contributed to the preparation and revision of the manuscript, and read and approved its final version.
